# Supplementary material for: Salmonella cancer therapy metabolically disrupts tumours at the collateral cost of T cell immunity
Source: EMBO Mol Med. 2024 Nov 18;16(12):4. doi: 10.1038/s44321-024-00159-2 (PMC11628626; doi:10.1038/s44321-024-00159-2)
Supplement: Supplementary file 1 — Appendix [file 44321_2024_159_MOESM1_ESM.pdf]

Appendix – Table of contents

Salmonella cancer therapy metabolically disrupts tumours at the collateral cost of T cell immunity

Page 2: Appendix Figure S1: In vivo gating strategy

Page 3: Appendix Figure S2: Timer Expression Correlates with PD-1 Expression

Page 4: Appendix Figure S3: Ex vivo tumour fragment gating strategy

Page 5: Appendix Table S1: Exact p values

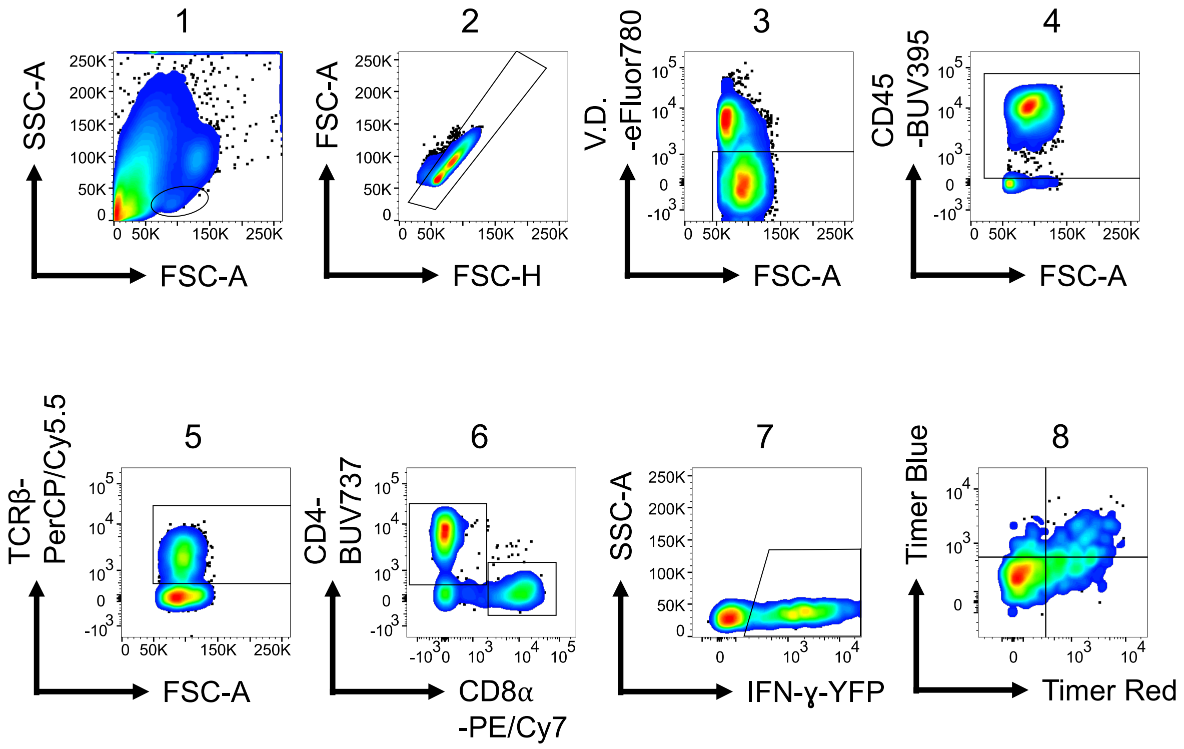

Appendix Figure S1: In vivo gating strategy

A representative gating strategy for analysis of TILs from the CAC model. Cells were gated by size to exclude epithelial debris (1)® single cells (2) ® live cells (3) ® leukocyte lineage (4) ® T cells (5) ® CD4 or CD8 (6) ® IFN-g positive (7) ® *Nr4a3*-Timer expression.

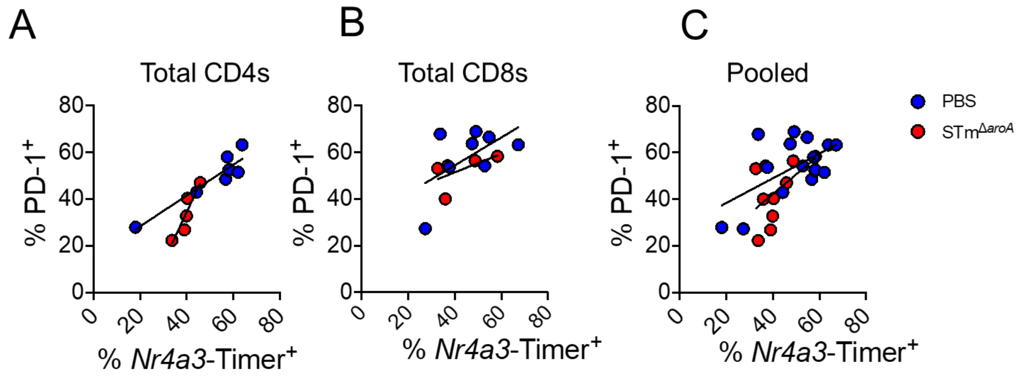

Appendix Figure S2: Timer Expression Correlates with PD-1 Expression

Correlation between PD-1 expression and *Nr4a3*-Timer positivity. TILs were analysed from digested primary tumours and total CD4s (A), CD8s (B) or pooled T cells (C) were analysed for correlation between PD-1 positivity and *Nr4a3*-Timer positivity. Linear regression was applied to both data sets; line depicts best fit of the regression. Each data point represents a single mouse.

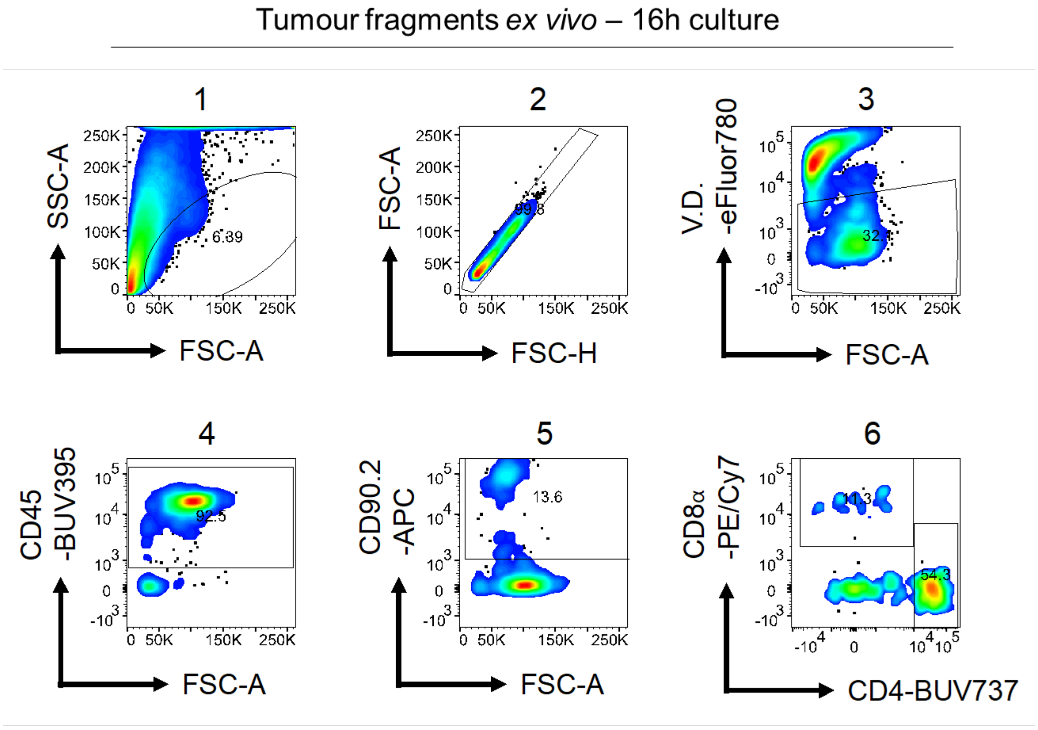

Appendix Figure S3: Ex vivo tumour fragment gating Strategy

(A) Representative gating strategy depicting analysis of TILs that had been cultured *ex vivo* in Matrigel for 16h after dissection. Cells were stimulated with a-CD3/a-CD28 antibodies (1 mg/mL and 5 mg/mL, respectively) for 16h and then processed for flow cytometry. Cells were gated by size to exclude epithelial debris (1)→ single cells (2) → live cells (3) → leukocyte lineage (4) → T cells (5) → CD4 or CD8.

Appendix Table S1: Exact P Values

Figure	Condition	Pval
**Figure 1b**	**CD4**	
	Spleen IFNg %	2,620E-06
	Spleen IFNg MFI	1,805E-07
	mLN IFNg MFI	6,700E-09
	Tumour IFNg%	7,890E-07
	Tumour IFNg MFI	4,300E-07
	**CD8**	
	Spleen IFNg %	7,410E-05
**Figure 1c**	**CD4**	
	Spleen	1,251E-06
	mLN	9,250E-07
	Tumour	6,570E-08
**Figure 3c**	all	1,800E-14
**Figure 3d**	**CD4 Timer Blue**	
	24hr	
	HK STmΔaroA vs. Live STmΔaroA	6,854E-07
	48hr	
	Non-treated vs. Live STmΔaroA	1,290E-13
	HK STmΔaroA vs. Live STmΔaroA	1,990E-13
	**CD4 Timer Red**	
	48hr	
	Non-treated vs. Live STmΔaroA	7,930E-10
	HK STmΔaroA vs. Live STmΔaroA	1,143E-09
	**CD8 Timer Blue**	
	24hrs	
	Non-treated vs. HK STmΔaroA	1,543E-05
	Non-treated vs. Live STmΔaroA	2,521E-09
	HK STmΔaroA vs. Live STmΔaroA	1,670E-13
	48hrs	
	Non-treated vs. Live STmΔaroA	1,800E-14
	HK STmΔaroA vs. Live STmΔaroA	1,800E-14
	**CD8 Timer Red**	
	24hrs	
	HK STmΔaroA vs. Live STmΔaroA	1,782E-05
	48hrs	
	Non-treated vs. Live STmΔaroA	5,633E-11
	HK STmΔaroA vs. Live STmΔaroA	3,245E-11
**Figure 3e**	CD4 % IFNg 48hrs	
	Non-treated vs. Live STmΔaroA	6,206E-06
	HK STmΔaroA vs. Live STmΔaroA	2,218E-05
	CD8 %IFNg 24hrs	
	Non-treated vs. Live STmΔaroA	2,085E-05
	HK STmΔaroA vs. Live STmΔaroA	2,476E-09

	CD8 % IFNg 48hrs	
	Non-treated vs. Live STmΔaroA	6,210E-13
	HK STmΔaroA vs. Live STmΔaroA	1,845E-12
**Figure 3g**	Non-treated	7,408E-11
	HK STmΔaroA	8,144E-11
**Figure 5d**	CD4 Glut1	5,258E-06
	CD8 Glut1	8,469E-05
**Figure 6d**	NFAT1+	
	NT	3,954E-06
	STmΔaroA	5,139E-06
**Figure 7d**	**24 hrs**	
	Non-treated vs. STmΔaroA	8,527E-06
	STmΔaroA vs. STmΔaroA/ΔansB	5,530E-05
	**48 hrs**	
	Non-treated vs. STmΔaroA	6,714E-08
	STmΔaroA vs. STmΔaroA/ΔansB	1,660E-06
**Figure 7e**	CD8s arrested	
	Non-treated vs. STmΔaroA	4,003E-05
	STmΔaroA vs. STmΔaroA/ΔansB	1,596E-05
**Figure 8c**	Non-treated vs. STmΔaroA	1,163E-05
	STmΔaroA vs. STmΔaroA + 10 mM Asn	7,667E-04
	STmΔaroA vs. STmΔaroA/ΔansB	2,425E-03
	Non-treated vs. STmΔaroA/ΔansB	4,902E-02
**Figure 8d**	Non-treated vs. 250 uM	2,044E-02
	Non-treated vs. 500 uM	1,921E-06
**Figure 8f**	Non-treated vs. 100 uM	1,129E-04
	Non-treated vs. 250 uM	6,769E-06
	Non-treated vs. 500 uM	5,876E-06
**Figure 8k**	In vitro	
	PBS vs. STmΔaroA	3,600E-12
	STmΔaroA vs. STmΔaroA/ΔansB	2,920E-13
	Ex vivo	
	PBS vs. STmΔaroA/ΔansB	4,588E-06
	STmΔaroA vs. STmΔaroA/ΔansB	3,550E-07
**Figure EV3a**	TEM	9,200E-05
	TCM	4,600E-05
