# Supplementary figures and images for: Salmonella cancer therapy metabolically disrupts tumours at the collateral cost of T cell immunity
Source: EMBO Mol Med. 2024 Nov 18;16(12):4. doi: 10.1038/s44321-024-00159-2 (PMC11628626; doi:10.1038/s44321-024-00159-2)

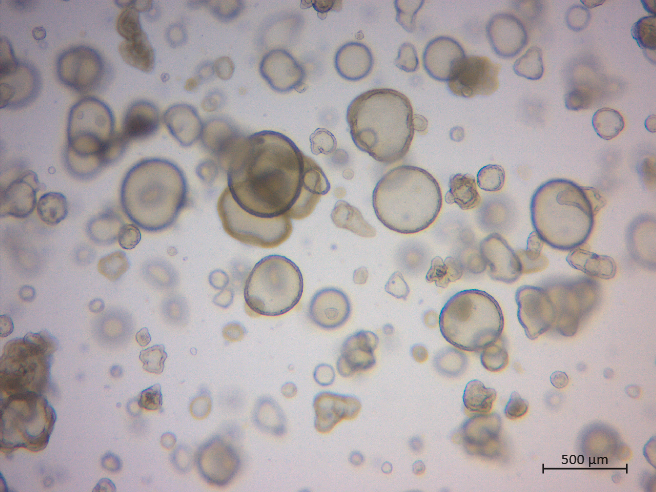

Supplement: Supplementary file 7 — Source data Fig. 8 [file 44321_2024_159_MOESM7_ESM.zip › Figure8/Figure8B/aroA+asn.tiff]

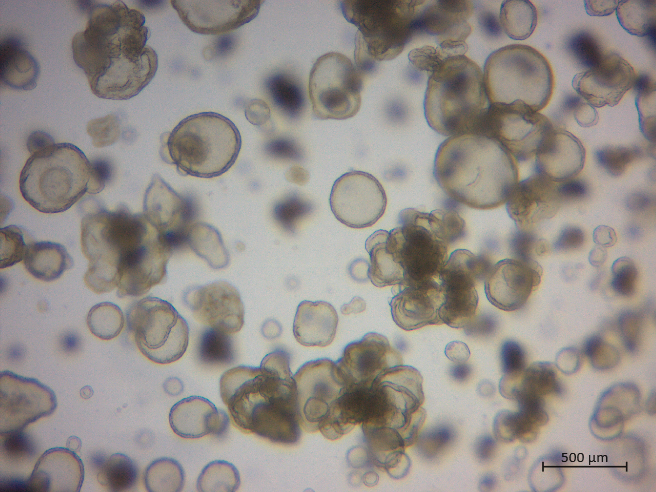

Supplement: Supplementary file 7 — Source data Fig. 8 [file 44321_2024_159_MOESM7_ESM.zip › Figure8/Figure8B/aroA.tiff]

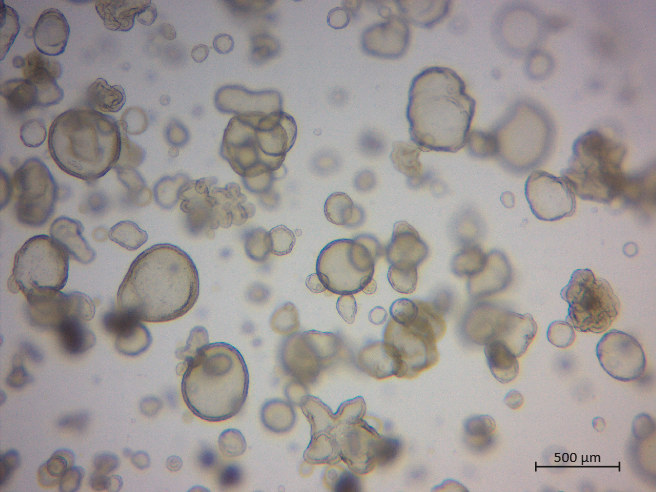

Supplement: Supplementary file 7 — Source data Fig. 8 [file 44321_2024_159_MOESM7_ESM.zip › Figure8/Figure8B/aroAansB.tiff]

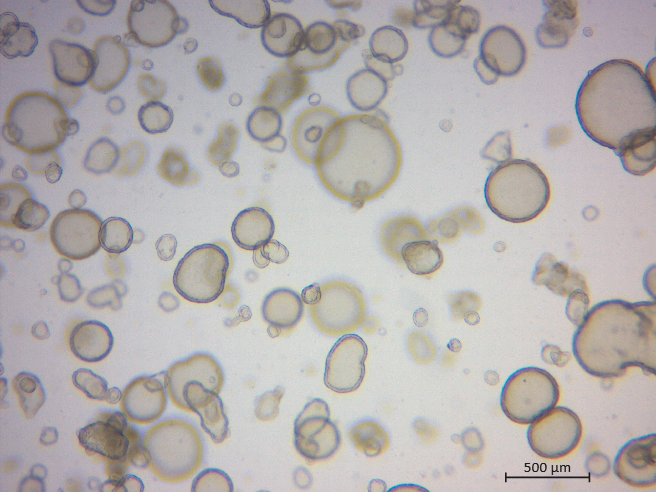

Supplement: Supplementary file 7 — Source data Fig. 8 [file 44321_2024_159_MOESM7_ESM.zip › Figure8/Figure8B/non-treated.tiff]
