# Supplementary material for: Salmonella cancer therapy metabolically disrupts tumours at the collateral cost of T cell immunity
Source: EMBO Mol Med. 2024 Nov 18;16(12):4. doi: 10.1038/s44321-024-00159-2 (PMC11628626; doi:10.1038/s44321-024-00159-2)
Supplement: Supplementary file 8 — Expanded View Figures [file 44321_2024_159_MOESM8_ESM.pdf]

Expanded View Figures

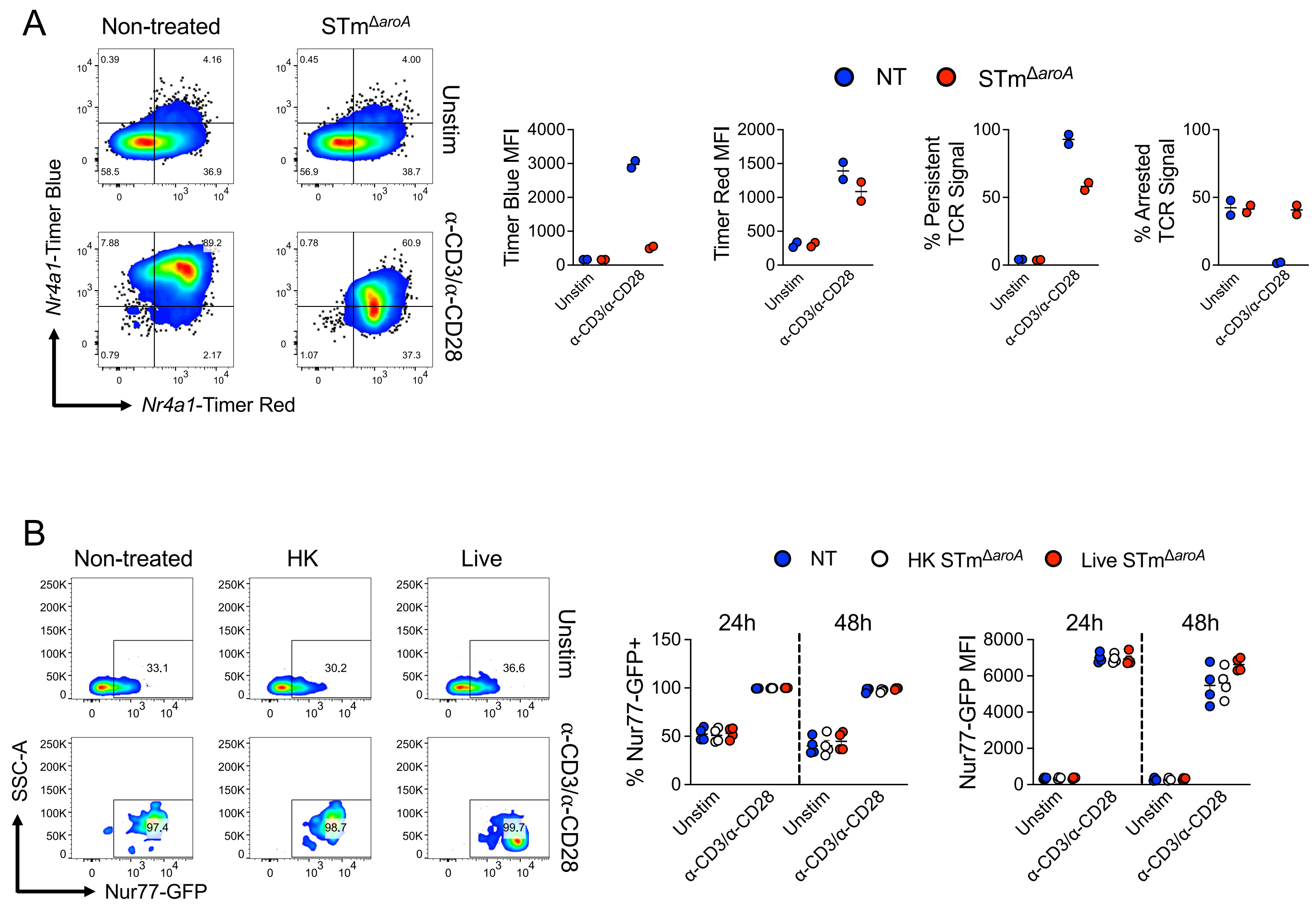

Figure EV1. Differential sensitivity of Timer and GFP-based TCR reporters in detecting arrested T cell activation.

TCM from NT or STm-infected tumour organoids was used to culture splenocytes from *Nr4a1*-Tempo (**A**) or *Nur77*-GFP (**B**) T cell reporter mice for 24 h (Tempo mice) or up to 48 h (Nur77 mice) after stimulation with α-CD3/α-CD28 (1 and 5 mg/mL, respectively). Data depict $n = 2$ (**A**) or $n = 3$ (**B**) mice.

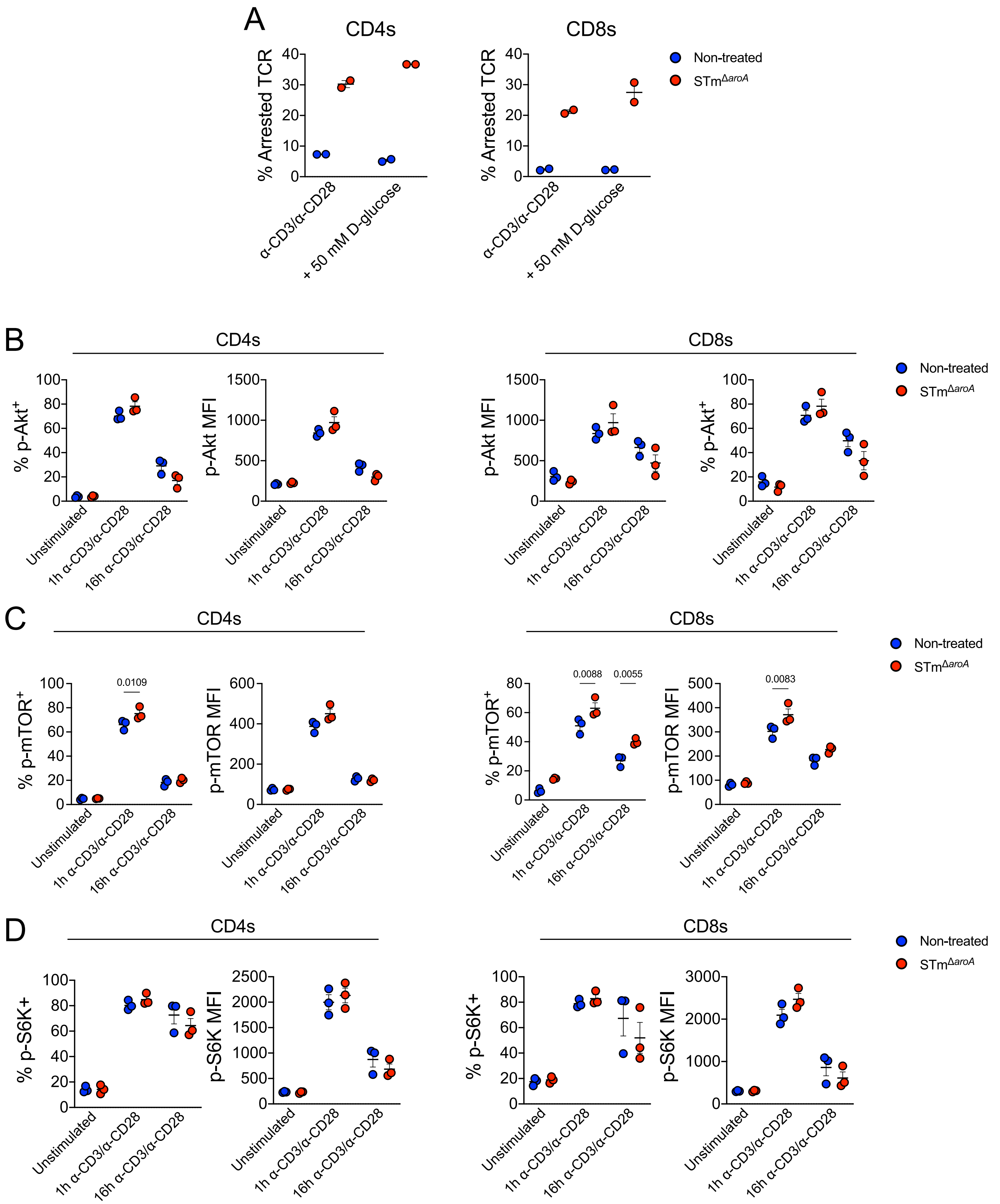
A
CD4s
CD8s
% Arrested TCR
α-CD3/α-CD28
+ 50 mM D-glucose
Non-treated
STm$^{\Delta aroA}$
B
% p-Akt^{+}
p-Akt MFI
C
0.0109
0.0088
0.0055
0.0083
% p-mTOR^{+}
p-mTOR MFI
D
% p-S6K+
p-S6K MFI
Unstimulated
1h α-CD3/α-CD28
16h α-CD3/α-CD28

© The Author(s)

◀ **Figure EV2. T cells activated in the presence of STm-infected tumours are unable to increase glucose uptake and show normal TCR signalling cascades.**

(**A**) Splenocytes were co-cultured with infected or non-treated tumours for 24 h in the presence of α-CD3/α-CD28 antibodies (1 and 5 µg/mL, respectively). During the final 6 h, a high dose of glucose (50 mM) was spiked into the culture in an attempt to reduce arrested TCR activation, as measured by *Nr4a3*-Timer Arrested TCR signal, i.e. *Nr4a3*-Timer Blue^{neg}Timer Red^{pos}. Cells were then tested for activation by flow cytometry. Data show two independent infections; each data point represents splenocytes tested with an independent tumour infection. (**B–D**) Quantification of PhosFlow signalling pathways as shown by representative plots in Fig. 6E. Cells were stimulated in NT and STm TCM for 1 or 16 h and processed for PhosFlow as previously described, showing p-Akt-S473 (**B**), p-mTOR-S2448 (**C**) and p70-S6K-T421/S424 (**D**). Bars depict means ± SEM. Statistical significance was tested by two-way ANOVA with Sidak's post-test (non-treated vs STm). Data were derived from $n = 3$ mice testing pooled TCM from two tumour infections.

© The Author(s)

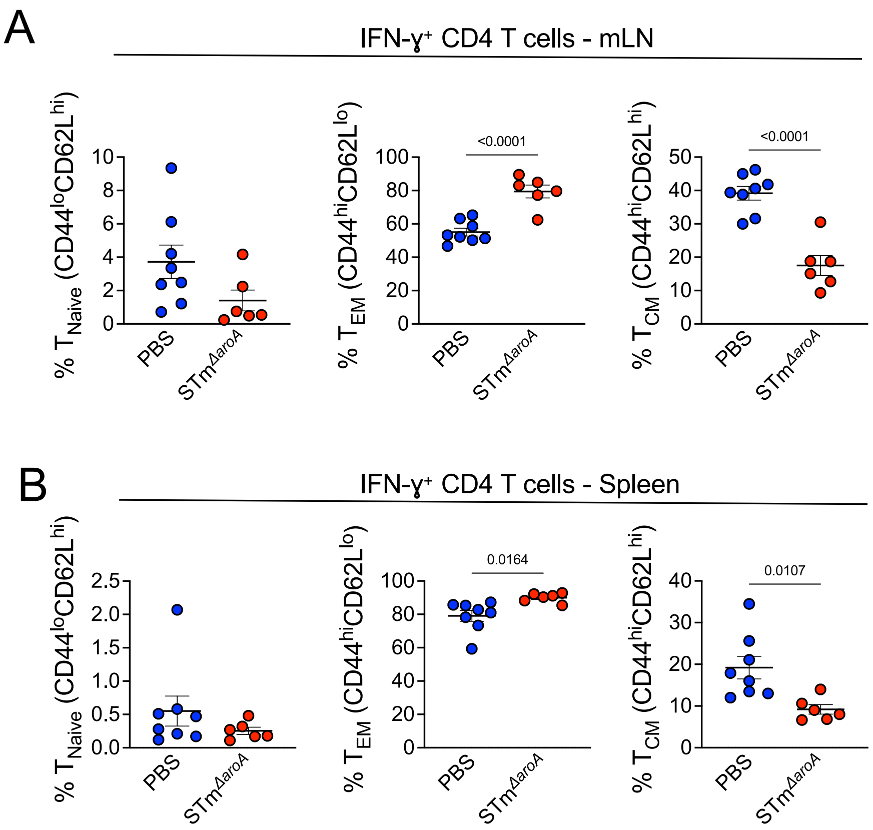

Figure EV3. Activated lymphatic T cells from mice infected with STm show a T_{EM}-bias.

Tumours were induced in mice using the CAC model, followed by two rounds of oral STm treatment as previously outlined for immunogenicity experiments. One week after the final dose, mice were culled and mLN and spleens were extracted, followed by preparation of single-cell suspensions and flow cytometry staining for T cell memory subsets within the IFN-γ⁺ CD4 (**A**) and CD8 T cell (B) populations. Cells were phenotyped based on the expression of CD44 and CD62L. Bars depict means ± SEM. Each data point represents one mouse, $n = 8$ NT, $n = 6$ *STm*$^{\Delta aroA}$. Statistical significance was tested by unpaired two-tail *t*-test.

 © The Author(s)

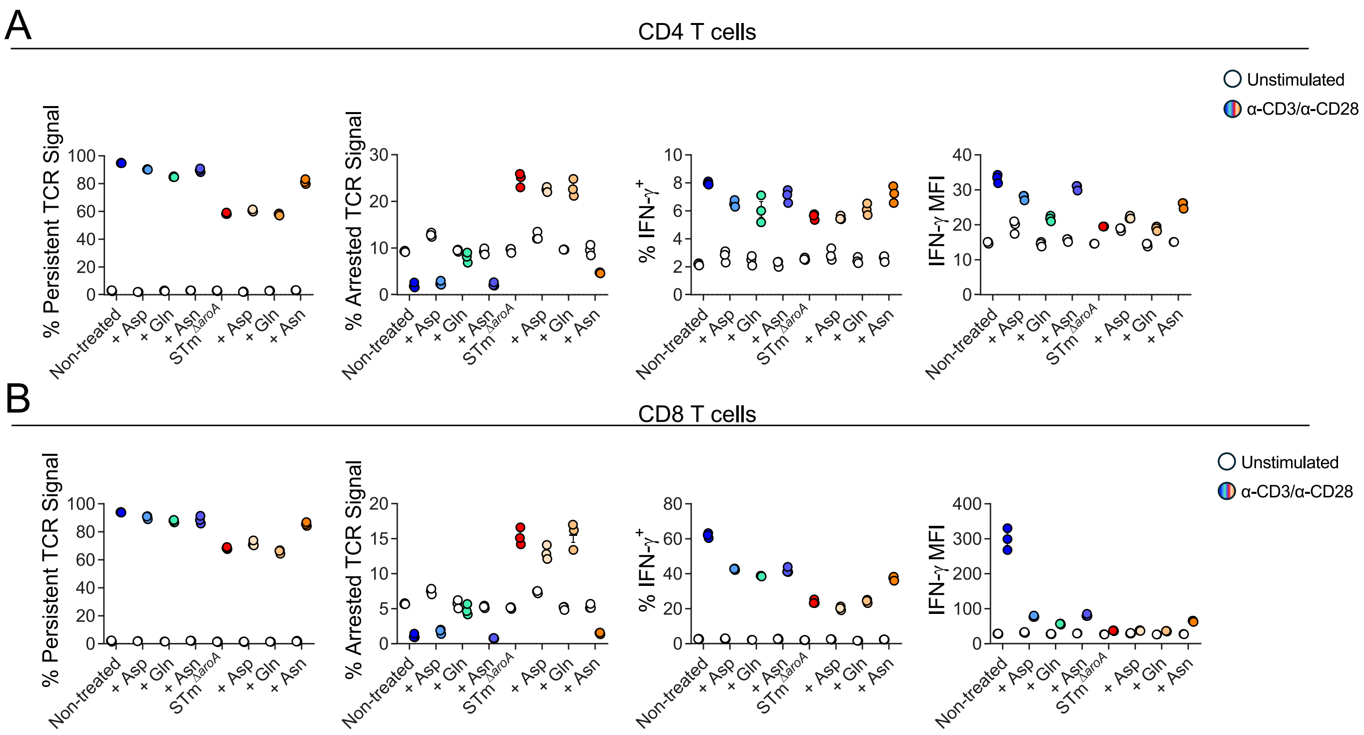

Figure EV4. Asparagine supplementation is able to restore T cell activation when cultured with TCM from infected tumours.

Splenocytes were cultured in TCM from either non-infected or infected-tumour organoids and activated for 24 h with α-CD3/α-CD28 antibodies (1 and 5 μg/mL, respectively). To some cultures, Asp/Gln/Asn were added (10 mM) at the beginning of the culture, or else a vehicle control (H_2O) was used. Various metrics of either *Nr4a3*-Timer, indicative of TCR signalling, or IFN-g expression were quantified by flow cytometry for CD4 (**A**) and CD8 (**B**) T cells. Data are from $n = 3$ mice, using pooled TCM from two tumour infections. Bars depict means ± SEM.

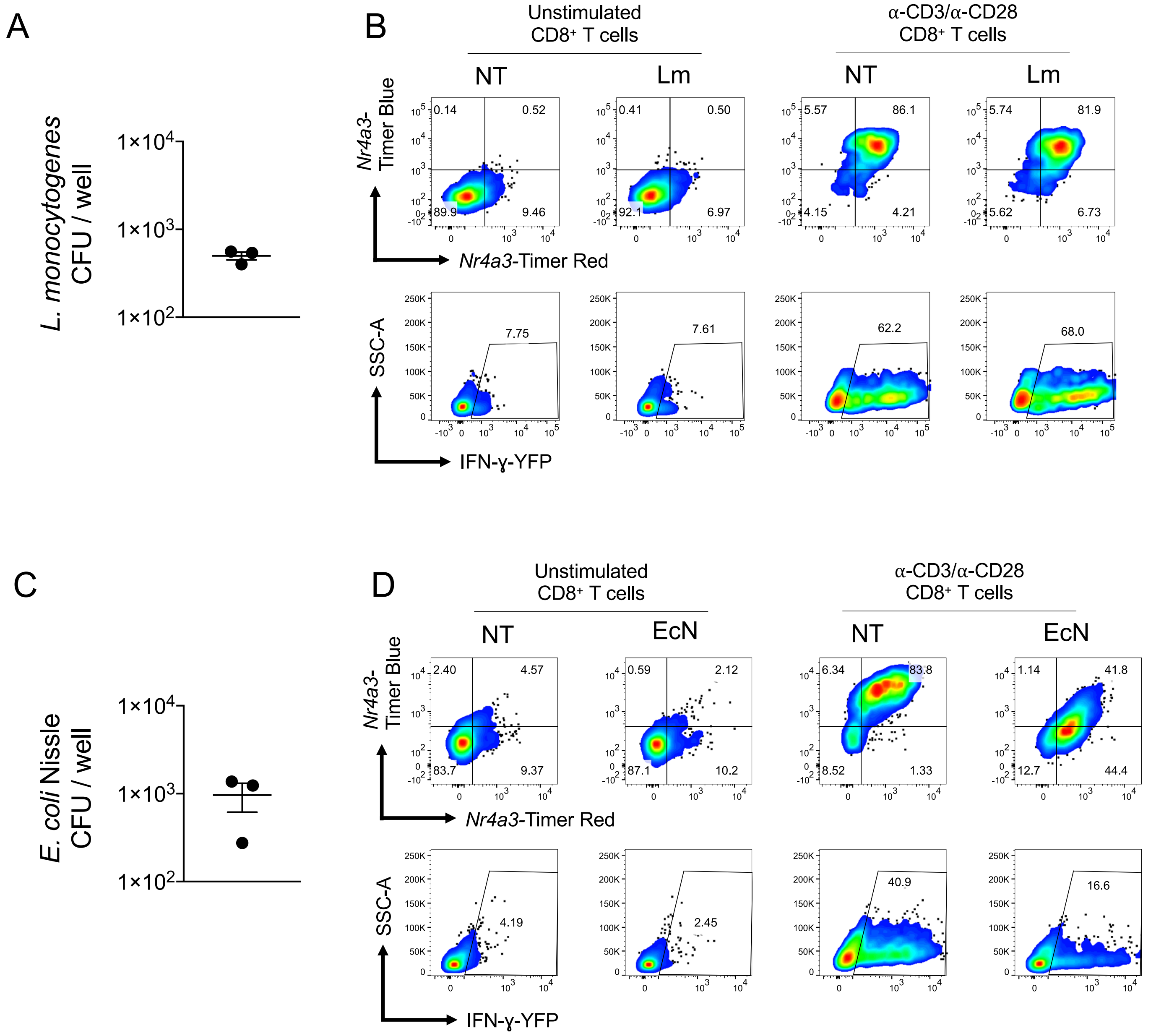

Figure EV5. Oncolytic bacteria possessing the high activity ASNase gene also suppress T cell Activation.

Tumour organoids were infected with either *L. monocytogenes* (Lm) or *E. coli* Nissle (EcN) and the TCM was used to stimulate splenocytes. (**A**) CFU assessment of Lm 24 hr after infection. Each dot represents an individual well of organoids, $n = 3$. (**B**) *Nr4a3*-Timer and IFN-γ expression of unstimulated or a-CD3/a-CD28 stimulated (1 and 5 mg/mL, respectively) CD8 + T cells from activated splenocytes cultured in Lm TCM. Data representative flow plots of $n = 3$ independent spleen donors. (**C**) CFU assessment of E.coli Nissle after 24 h infection. Each dot represents an individual well of organoids, $n = 3$. (**D**) *Nr4a3*-Timer and IFN-γ expression of unstimulated or a-CD3/a-CD28 stimulated (1 and 5 mg/mL, respectively) CD8 + T cells from activated splenocytes cultured in EcN TCM. Data representative flow plots of $n = 3$ independent spleen donors. Error bars depict SEM.

 © The Author(s)
